# Fucoidan-Containing, Low-Adhesive Siloxane Coatings for Medical Applications: Inhibition of Bacterial Growth and Biofilm Development

**DOI:** 10.3390/ma16103651

**Published:** 2023-05-10

**Authors:** Todorka G. Vladkova, Anna D. Staneva, Ivalina A. Avramova, Iliana A. Ivanova, Dilyana N. Gospodinova

**Affiliations:** 1 Department of Polymer Engineering, University of Chemical Technology and Metallurgy, 8 Kliment Ohridski Blvd., 1756 Sofia, Bulgaria; 2 Department of Silicate Technology, University of Chemical Technology and Metallurgy, 8 Kliment Ohridski Blvd., 1756 Sofia, Bulgaria; ani_sta@uctm.edu; 3 Institute of General and Inorganic Chemistry, Bulgarian Academy of Sciences, Acad. G. Bonchev Str., Block 11, 1113 Sofia, Bulgaria; iva@svr.igic.bas.bg; 4 Faculty of Biology, Sofia University “St. Kl. Ohridski”, 8 Dragan Tsankov Str., 1164 Sofia, Bulgaria; ilivanova@abv.bg; 5 Faculty of Electrical Engineering, Technical University of Sofia, 8 Kliment Ohridski Blvd., 1756 Sofia, Bulgaria; dilianang@tu-sofia.bg

**Keywords:** medical siloxane coatings, fucoidan-containing, surface characteristics, bacterial growth inhibition

## Abstract

The deposition of low-adhesive siloxane coatings is a current trend for the non-toxic control of bacterial growth and biofilm formation. Total elimination of biofilm formation has not been reported so far. The aim of this investigation was to study the ability of a non-toxic, natural, biologically active substance, such as fucoidan, to inhibit bacterial growth on similar medical coatings. The fucoidan amount was varied, and its impact on the bioadhesion-influencing surface characteristics, as well as on bacterial cell growth, was investigated. The inclusion of up to 3–4 wt.% brown algae-derived fucoidan in the coatings increases their inhibitory effect, more significantly on the Gram-positive bacterium *S. aureus* than on the Gram-negative bacterium *Escherichia coli*. The biological activity of the studied siloxane coatings was ascribed to the formation of a low-adhesive, biologically active surface top layer consisting of siloxane oil and dispersed water-soluble fucoidan particles. This is the first report on the antibacterial activity of fucoidan-containing medical siloxane coatings. The experimental results give reason to expect that relevantly selected, natural biologically active substances can be efficient in the non-toxic control of bacterial growth on medical devices and, as a result, medical device-associated infections.

## 1. Introduction

Medical device-associated infections due to bacterial growth and biofilm formation are major contributors to prolonged illnesses, the financial burden on patients, and increased mortality. Bacterial biofilm is implicated in almost 65% of microbial diseases and more than 80% of chronic infections [1]. The life in a biofilm increases sharply the microbial resistance to antibiotic and multi-drug treatments, which creates the need to look for new antimicrobial agents and approaches to inhibit bacterial growth on indwelling medical devices [2,3].

The development of low-adhesive material surfaces that prevent or make difficult the attachment and growth of microbial cells is a current trend in the non-toxic control of biofouling. Unfortunately, no modified or coated material surface has been reported to date that totally stops the development of medical biofilm [4,5]. The most important prerequisite for low bio-adhesion is high hydrophilicity, or high/superhydrophobicity, combined with a low elastic modulus of the material surface [6]. Therefore, siloxane elastomers combining low surface energy with a low elastic modulus and other advantages, such as good biocompatibility, transparency, etc., are preferable for the fabrication of medical devices and medical antimicrobial coatings [6].

Previously, marine anti-biofouling siloxane coatings have been developed that do not allow the attachment of macro-organisms and reduce marine biofilm formation, even on static immersed surfaces [7]. Their anti-biofilm performance was significantly improved by including antioxidants (combined or not with surfactant) in the coating composition, supposing a possible oxidative cross-linking of some microbial exopolymeric substances (EPSs) [8,9,10]. This led to the idea to try to improve the anti-biofilm performance of medical, low-adhesive siloxane coatings by exploring the anti-oxidative potential of some non-toxic natural products.

The replacement of antibiotics and toxic biocides with natural biologically active substances is the focus of many current investigations. However, their potential as anti-biofilm agents has not been fully investigated, although some of them show a proven, high-biological activity and are market-available [11,12]. One of them is fucoidan, considered as a promising antimicrobial component that could replace some currently used antibiotics. The name “fucoidan” does not represent a substance with a given structure. It is a heterogeneous group of water-soluble polysaccharide complexes whose main constituent is sulfated fucose. It builds up a linear backbone by alternating the (1 ⟶ 3) and (1 ⟶ 2), or (1 ⟶ 4) linkages and sulfated groups at the C4, C2, or C2, C3 positions, as presented in Figure S1 [13,14,15]. Fucoidan bioactivity varies depending on the seaweed source, its complex structure, molecular weight, monosaccharide composition, sulfate content and position, linkage mode, sequence of monosaccharide residues, substitution position, etc. [14]. Crude extracts with a wide range of biological activity (including antioxidant along with anti-inflammatory, anticoagulant, antitumor, antithrombotic, antivirus, immunomodulation, and some new) are commercially available as nutritional supplements [16,17]. Updated reviews [15,18] discuss the beneficial functions of fucoidan extracted from brown seaweed in human medicine because of its antioxidant, immune-modulating, anti-allergic, antitumor, antiviral, anti-inflammatory, and hepato-protective effects. Lately, Koik et al. demonstrated the binding abilities of low-molecular fucoidan derivatives to SARS-CoV-2 spike proteins [19].

The reports on the anti-biofilm activity of fucoidan are scare, divers for extracts from different seaweeds and their efficiency against different bacterial biofilms. Chmita et al. [20] report a negligible effect of fucoidan from *Laurus hobilis* on biofilm formation, although a good activity against five Gram-negative and Gram-positive bacterial strains. A notable antimicrobial activity of fucoidan from *Fucus vesiculosus* against dental plaque planktonic bacteria (*Streptococcus mutants* and *Streptococcus sobrinus*) and the suppression of complete biofilm formation, but no eliminative effect on the already completed biofilm, are reported by Jun et al. [21]. Enhanced resistance to plasma deposition and growth of *Staphylococcus aureus* pathogens on sulfated-fucoidan hydrophilic antiadhesive polydimethylsiloxane-covered implants was found by Mohan et al. [22]. According to Achmad et al. [23], fucoidan derived from brown seaweed could be utilized as an antimicrobial and anti-biofilm agent in the prevention of early dental caries by inhibiting dental biofilm formation. A fucoidan-loaded zeolite imidazole framework has been demonstrated to have antimicrobial activity against *Bacillus subtilis*, *Staphylococcus aureus*, *Klebsiella pneumonia*, and *Escherichia coli*, as well as to disturb effectively the methicillin-resistant *S. aureus* and *E. coli* biofilm architecture [24]. Tang et al. [25] present excellent in vitro inhibiting effects of fucoidan-derived carbon dots on *E. faecalis* biofilm and infected dental tubules for the treatment of persistent endodontic infections.

The antioxidant and antibacterial activities of fucoidan derived from seaweed against planktonic cells are well known, but its inhibitory effect on bacterial growth and biofilm development on material surfaces remains insufficiently studied and practically not explored [11,26]. Reports on the effect of fucoidan-containing medical siloxane coatings on bacterial cell growth and biofilm formation cannot be found. Thus, the aim of this study includes the preparation and characterization of fucoidan-containing medical low-adhesive siloxane coatings, as well as an evaluation of their antibacterial action, varying both the fucoidan amount (0–8 wt.%) and time of inoculation (24 h and 48 h).

## 2. Materials and Methods

The influence of the fucoidan amount and inoculation time on Gram-negative and Gram-positive bacterial cell growth was evaluated by the most probable number living cells test using glass samples covered with the studied low-adhesive siloxane coatings. Furthermore, the bioadhesion-influencing, surface physical–chemical, and physical–mechanical parameters were estimated to be correlated with the initial cell interactions.

### 2.1. Coating Compositions

The coating compositions used in this investigation consisted of two components: room temperature vulcanizing, medical grade, platinum silicone elastomer (MED-4210, Factor II, Incorporated, Lakeside, OK, USA); 10 wt.% siloxane oil (1000 cP, DMS-T31, Gelest, Morrisville, PA, USA); and 0–8 wt.% fucoidan, extracted from brown seaweed *Fucus vesiculosus* (Sigma Aldrich, St. Louis, MO, USA). The coating compositions were prepared by mixing [7].

### 2.2. Coated Test Samples

Glass plates (10 × 10 × 2 mm) were spin-coated (at 400 min^−1^) with a primer, consisting of ethyl-triacetoxysilane (50 wt.% toluene solution) and a catalyst (3 wt.% dibutyltindilaurate) to provide good adhesion of the coating to the glass surface. The primed dry glass plates were then spin-covered with a corresponding coating composition under the same conditions. Prior to testing, the covered test samples were kept under ambient room conditions for at least 30 days to be completely cross-linked. The thickness of the dry coatings was 200–220 mkm as measured by a stereo microscope Leica MZ16 FA (Leica, Wetzlar, Germany).

The numbering of the test samples was as follows:

Sample (1)—Control: glass, covered with siloxane coating, does not contain fucoidan;
Sample (2)—Glass, covered with siloxane coating, containing 1 wt.% fucoidan;
Sample (3)—Glass, covered with siloxane coating, containing 2 wt.% fucoidan;
Sample (4)—Glass, covered with siloxane coating, containing 3 wt.% fucoidan;
Sample (5)—Glass, covered with siloxane coating, containing 5 wt.% fucoidan;
Sample (6)—Glass, covered with siloxane coating, containing 8 wt.% fucoidan.

### 2.3. Water Contact Angle (WCA) and Surface Energy (Ec)

The contact angle-measuring instrument Easy Drop (Kruss, Hamburg, Germany) was employed for the static contact angle measurements (angle resolution ± 0.10°) using three liquids with known surface tension: water, ethylene glycol, and hexadecene. The surface energy (E) was calculated according to Fowkes’ method [27].

### 2.4. Atom Force Microscopy (AFM)

An Easyscan 2 apparatus equipped with a Pointprobe Contr-10 silicone SPM sensor (Nanosurf, Liestal, Switzerland; dimensions 2 × 450 × 50 µm^3^) operating in a contact mode was employed to obtain plane and 3D images of the investigated dry surfaces. Vicker’s Diamond Pyramid (Nanosurf, Liestal, Switzerland) with a pike angle of 136° was used for all the measurements at room temperature, with a loading speed of 0.250 mN/s.

### 2.5. Depth-Sensing Indentation (DSI)

A dynamic Ultra Micro-Hardness Meter DUH-211 S (Shimatzu, Kyoto, Japan) was used to evaluate the indentation hardness (HIT), Vicker’s hardness (VIH), and indentation elastic modulus (EIT) under the following conditions: test force of 0.55 mN; loading speed of 6.5 (0.0250) mN/s; and penetration depth of 30 nm.

### 2.6. X-ray Photoelectron Spectroscopy (XPS)

The XPS analysis was performed on a Kratos AXIS Supra spectrometer with a mono-chromatic Al X-ray source under a vacuum deeper than 10^−8^ Pa at a 90° take-off angle. Each analysis started with a survey scan from 0 to 1200 eV and a pass energy of 160 eV at steps of 0.5 eV with 1 sweep. For the high-resolution analysis, the number of sweeps was increased, and the pass energy was lowered to 20 eV at steps of 100 meV. The C1s photoelectron line at 285 eV was used for the calibration of the recorded spectra.

### 2.7. In Vitro Antibacterial Activity

#### 2.7.1. Test Bacterial Strains

The test microbial strains, both Gram-negative bacterium *Escherichia coli*, ATCC 25922, and Gram-positive bacterium *Staphylococcus aureus*, ATCC 25923, were provided by the National Bank of Microorganisms and Cell Cultures (NBIMCC), Bulgaria.

#### 2.7.2. Cell Growth Inhibition

The most probable number of living cells (MPN) test [28] was used to evaluate the bacterial cell growth inhibition above the coated test samples, as it was made earlier [29].

The test cultures were grown in a nutrient broth (Conda, Madrid, Spain) on a rotary shaker (Rotamax, Haiger, Germany, 180 RPM) at 37 °C overnight followed by the sub-culturing of bacteria the next day. A total of 0.1 mL was put in 5 mL of fresh medium at the same conditions on a shaker until the optical density of 0.5 MacFarland was achieved, as detected by a Grant-bio DEN-1 densitometer. The thin film coated glasses of 2.0 × 2.0 cm were placed on the bottom of each well of sterile 6-well plates and sterilized by UV irradiation for 30 min. A 3.00 mL bacterial suspension was used for the inoculation of the thin films in the 6-well plates (Corning Costar). The inoculated films were kept in an incubator at 37 °C for 24–48 h and shaken at 50 RPM to imitate real conditions and allow the formation of bacterial films on the coatings. After inoculation, samples of 0.1 mL were taken from the bacterial suspension over the coatings at the first hour. Decimal dilutions were prepared and Petri dishes with sterile nutrient agar (Conda, Madrid, Spain) were inoculated to enumerate the starting microbial quantity.

At 24 and 48 h, the coated glasses were taken from the plate, put in a new sterile 6-well plate, and rinsed with 1 mL of sterile saline (0.85% NaCl). Then, 0.1 mL of rinsed liquid was taken to prepare the decimal dilution, and 0.1 mL from each dilution was inoculated in a Petri dish with a solid nutrient medium to determine the bacterial quantity. Each time the coated glass was put in a new sterile well, it was rinsed with 1 mL of saline, using a total of 3 mL saline. After the last rinsing, 0.1 mL from the 3rd rinsing of saline aliquot was taken for preparing the decimal dilution and enumeration of bacterial cells. The decimal dilutions were used for the inoculation (100 μL) of the nutrient agar for counting the surviv bacteria. The Petri dishes were cultivated in the dark at 37 °C, and the number of bacterial colonies was counted at 24 and 48 h. The formula for the cell number determination of the bacterial suspension was:

N = A × 10^n^/0.1,

where

N is the number of bacteria in the rinse liquid;
A is the number of counted colonies in a Petri dish from a decimal dilution;
n is the relevant number of decimal dilutions used to inoculate the Petri dish; and
0.1 is the quantity of inoculum in every Petri dish.

All the results are the average of 3 measurements.

#### 2.7.3. Fluorescent Microscopy Observation

Fluorescence microscopy was used for the observation of the biofilm on the glass samples covered with a siloxane coating, either containing or not containing fucoidan. The coated test samples were stained with diamino-2-phenyl-indol (DAPI, Molecular Probes, Invitrogen, CA, USA) and kept for 10–15 min in the dark for coloration. The samples were either unrinsed or rinsed in 3 mL of saline and observed using a fluorescence microscope (Leica DM 5500B, Leica Microsystems, Vienna, Austria) equipped with an integrated camera.

## 3. Experimental Results and Discussion

### 3.1. Surface Characteristics

The main bioadhesion-influencing surface characteristics of the coated glass samples, estimated before immersion in the microbial suspension, are presented in Table 1.

The data in Table 1 show that all the surface characteristics (WCA, E, E_d_, E_p_, HMV, HIT, and EIT) of the studied composition coatings are influenced by the presence of fucoidan.

With a WCA (measured at the initial time) varying by the fucoidan amount but above 105° (104.7° ± 4.17° up to 120.2° ± 0.22°) (Table 1, row 1), the studied coatings are superhydrophobic, which is a prerequisite for low bioadhesion [30,31]. An exception is sample 6, which is hydrophobic (WCA of 99.7° ± 2.28°). The WCA of samples 2–4, containing 1–3 wt.% fucoidan (Table 1, row 1), increases compared to the control without fucoidan (Table 1, sample 1) simultaneously with a slight increase in the surface roughness, Ra (from 0.139 nm up to 0.918 nm) and Rq (from 0.217 nm up to 1.316 nm) (Table 1, rows 9 and 10). The WCA decreases to 99.7° ± 2.28° when the fucoidan content is 5 wt.% and above simultaneously with a sharp increase of the surface roughness, Ra (up to 35.911 nm) and Rq (up to 40.213 nm), respectively (Table 1, samples 5 and 6). The surface energy, E, and its dispersive (E_d_) and polar (E_p_) components demonstrate deviations corresponding to those of the contact angle (Table 1, rows 3, 5, and 7).

Remaining above 90°, the WCA (Table 1, row 2) measured after 120 s decreases compared to that measured at the initial time (Table 1, row 1) for all the studied coatings (Table 1, samples 1–6), indicating the formation of a hydrophobic, water diffusion-allowing, vulcanization network. The formation of a vulcanization network that allows diffusion of liquids is confirmed by the wetting kinetics, expressed as a contact angle with water, ethylene glycol, and hexadecane (Figure S2). The corresponding contact angles with all three liquids decrease with the progression of time, more sharply in around the first 50 s. Considering that fucoidan is water-soluble and could be partially extracted in 120 s, the alterations in the WCA measured after 120 s compared to that measured at the initial time, and the respective surface energy, are not a surprise [14]. These alterations indicate that the newly developed coatings could be not only killing on contact but also delivering fucoidan as an antimicrobial agent.

The surface physical–mechanical parameters: dynamic Vicker’s hardness (HMV), indentation hardness (HIT), and indentation elastic modulus (EIT), presented in the last three rows of Table 1, demonstrate slight deviations for samples 2, 3, and 4 (containing 1 wt.%, 2 wt.%, or 3 wt.% fucoidan) compared to sample 1 (control, without fucoidan). When the fucoidan content is higher (i.e., 5 wt.% or 8 wt.%), the HMV, HIT, and EIT significantly increase (Table 1, samples 5 and 6). The observed alterations of the physical–mechanical parameters confirm the supposition about the specific vulcanization network formation in the presence of fucoidan. It probably influences the WCA and the free surface energy (E).

### 3.2. XPS

**Figure 1:**
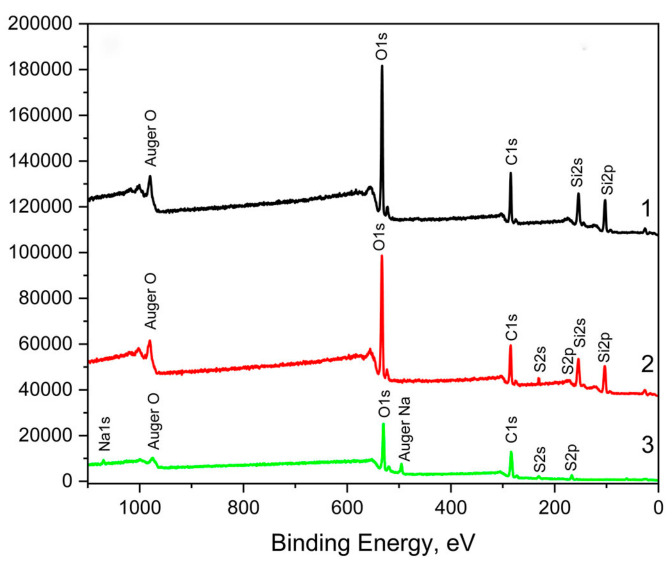
Survey scan spectra of vulcanized siloxane coatings: curve 1—without fucoidan; curve 2—containing 5 wt.% fucoidan; curve 3—fucoidan.

The results of the XPS analysis are presented in Figure 1 and Figure S3.

In the survey spectrum of the polysiloxane matrix (Figure 1, curve 1) are visible three peaks, detectable by the XPS elements: C, O, and Si, part of the following functional groups of the cross-linked polysiloxanes: -OCH_3_, -OH, and –Si–O–Si-. In the survey spectrum of the fucoidan (Figure 1, curve 3) are visible three peaks, detectable by the XPS elements: C, O, and S, which are part of the following functional groups: -OSO_3_^−^, -C–O-C, -OH, -CH_3_ and, in the six-atom carbon/oxygen rings containing two oxygen atoms, characteristic of polysaccharides.

In the survey spectrum of the sample containing 5 wt.% fucoidan (Figure 1, curve 2) are visible peaks of the elements C and O (originating from both the polysiloxane and fucoidan), as well as of Si (originating from the polysiloxane), and S (originating from the fucoidan). No additional, altered, or replaced peaks are observed in the survey scan spectrum of the sample containing 5 wt.% fucoidan (Figure 1, curve 2) compared to the spectra of the cross-linked siloxane elastomer (Figure 1, curve 1) and the fucoidan (Figure 1, curve 3). This indicates a lack of chemical interaction between the polysiloxane and the fucoidan, which is confirmed by the comparison of the deconvoluted C1s, O1s, Si2p, and S2p peaks presented in Figure S3. All of the deconvoluted C1s, O1s, Si2p, and S2p peaks of the coating with 5 wt.% fucoidan (Figure S3, curves 2) contain the carbon, oxygen, silica, and sulfur components, characteristics of the functional groups of the cross-linked siloxane elastomer (Figure S3, curves 1) and the fucoidan (Figure S3, curves 3).

The results of the XPS analysis give reason to assume that the fucoidan particles are dispersed in the elastomer matrix containing non-reactive siloxane oil because of the lack of chemical interaction between them.

### 3.3. Cell Growth Inhibition

**Figure 2:**
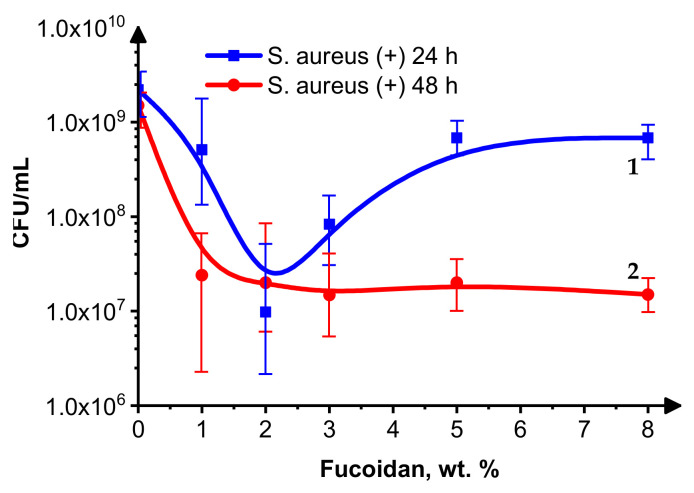
*S. aureus* growth inhibition in the presence of 0–8 wt.% fucoidan in a siloxane low-adhesive coating 24 h (curve 1) and 48 h (curve 2) after inoculation.

**Figure 3:**
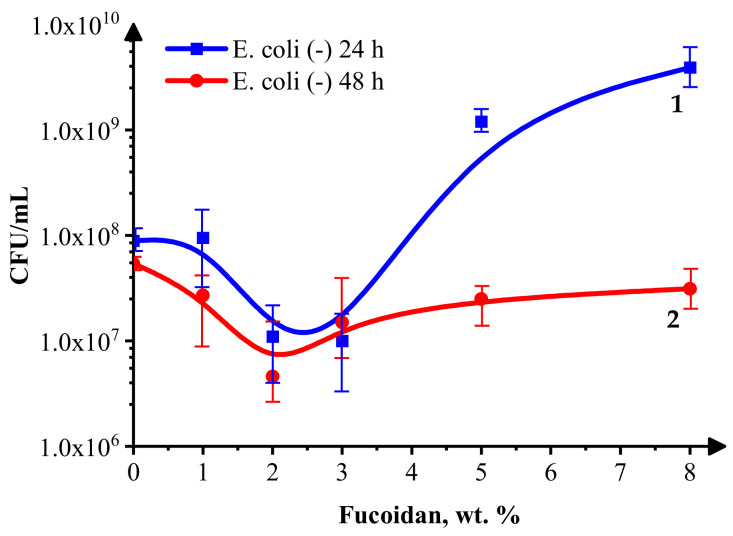
*E. coli* growth inhibition in the presence of 0–8 wt.% fucoidan in siloxane low-adhesive coating 24 h (blue curve) and 48 h (red curve) after inoculation.

The results of the *Staphylococcus aureus* and *Escherichia coli* growth inhibition by the fucoidan-containing medical siloxane coatings are presented in Figure 2 and Figure 3, respectively.

Figure 2 demonstrates that the activity of the fucoidan-containing siloxane coatings toward the Gram-positive bacterium *S. aureus* depends on the fucoidan content and the inoculation time. It is interesting that, for 24 h inoculation (Figure 2, curve 1), the most probable number (MPN) of living *S. aureus* cells decreases when the fucoidan concentration is below 2 wt.%. Except for the sample without fucoidan, above this concentration, the MNP of living *S. aureus* cells increases, probably due to the delivery of a higher fucoidan amount, which kills more bacterial cells, forming a new interface. The last one is made by non-growing cells, which are less vulnerable to a variety of antimicrobial agents [32] and make difficult the diffusion of the fucoidan particles to the *S. aureus* cell suspension. At 48 h post-inoculation, the MPN of living cells for all the fucoidan-containing samples is below that for the control sample without fucoidan and almost equal for all of them (Figure 2, curve 2), which indicates their moderate bacteriostatic effect on *S. aureus.*

Figure 3 demonstrates the fucoidan content and inoculation time-dependent antibacterial activity of the same siloxane coatings against the Gram-negative bacterium *E. coli*.

The MPN of living *E. coli* cells 24 h after inoculation (Figure 3, curve 1) decreases with the fucoidan concentrations up to about 3 wt.% and increases to a higher level, except the value for the control sample without fucoidan (likely assisting the growth of the *E. coli* cells). Perhaps the dead *E. coli* cells act as a food source for the living ones. The MPN of living *E. coli* cells at all the fucoidan concentrations (0–8 wt.%) is lower after 48 h than 24 h after inoculation (Figure 3, curve 2 is below curve 1), indicating a bacteriostatic effect of the studied coatings, most probably due to their low adhesiveness, which makes difficult the cell attachment and differentiation. The slight differences in the behavior of *S. aureus* and *E. coli* cells in contact with the studied coatings could be connected to their specificity, such as, for example, differences in their surface and other properties (*E. coli* has a more negatively charged and less soft surface than *S. aureus*).

The effect of the studied medical, low-adhesive siloxane coatings on Gram-positive *S. aureus* and Gram-negative *E. coli* bacterial growth 24 and 48 h after inoculation is moderate (the MPN of living cells decreases by 1–2 orders), and it is most pronounced at fucoidan concentrations of 1–3 wt.% for both *S. aureus* and *E. coli*. The number of surviving *E. coli* and *S. aureus* cells is slightly lower at 48 h than at 24 h after inoculation, indicating a bacteriostatic effect of these coatings. The increased superhydrophobicity (Table 1, row 1), combined with an insignificant alteration of the physical–mechanical parameters: dynamic Vicker’s hardness (HMV), indentation hardness (HIT), and indentation elastic modulus (EIT) (Table 1, the last three rows) at the relatively low fucoidan-loading levels of samples 2–4 (1 wt.%, 2 wt.%, and 3 wt.%), contributes to decreased bioadhesion and biofilm formation. Higher fucoidan-loading levels (5 wt.% and 8 wt.%) in samples 5 and 6, respectively, lead to a significantly increased EIT combined with decreased hydrophobicity, both known to negatively impact the antimicrobial performance [33].

### 3.4. Fluorescence Microscopy

**Figure 4:**
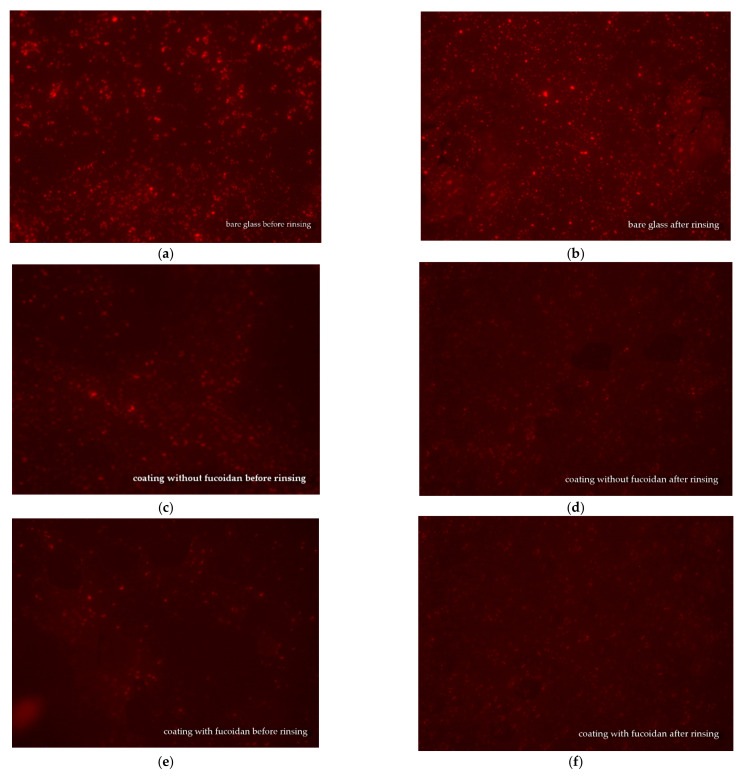
Fluorescence microscopy pictures (×400) of *S. aureus* biofilm on bare glass (**a**,**b**) and *S. aureus* colonies on siloxane coating without fucoidan (**c**,**d**), or on siloxane coating containing 3 wt.% fucoidan (**e**,**f**), before rinsing (**a**,**c**,**e**) and after rinsing in 3 Ml saline (**b**,**d**,**f**).

Fluorescence microscopy was used for the observation of the *S. aureus* and *E. coli* biofilms on glass samples covered with medical siloxane coatings, containing or not containing fucoidan. The samples were either not rinsed or rinsed in 3 mL of saline and then observed to evaluate the impact of the fucoidan on the antiadhesive properties of the studied siloxane coatings. The fluorescence microscopy pictures of *S. aureus* biofilm on bare glass and *S. aureus* colonies on siloxane coatings, both without fucoidan or containing fucoidan, before and after rinsing in saline, are presented in Figure 4.

A comparison of images (a) and (b) in Figure 4 demonstrates that the *S. aureus* biofilm formed on bare glass is almost the same before and after rinsing. Before rinsing out, *S. aureus* colonies are seen onto the siloxane coatings, both does not containing, Figure 4c and containing fucoidan, Figure 4e that are smaller number and smaller size at the fucoidan containing coating, Figure 4 e. The last one demonstrates the fucoidan contribution to the antibacterial activity of the studied siloxane coatings. After rinsing in saline, the *S. aureus* colonies on the siloxane coating without fucoidan are smaller, Figure 4d, as compared to those on the same coating before rinsing, Figure 4c, because of the easy detachment of a part of the *S. aureus* cells from this low adhesive surface. A similar picture is observed on the fucoidan-containing coating: after rinsing: the *S. aureus* colonies get smaller, Figure 4f, than those before rinsing, Figure 4e. This demonstrates that fucoidan has no impact on the adhesiveness of the studied coatings.

**Figure 5:**
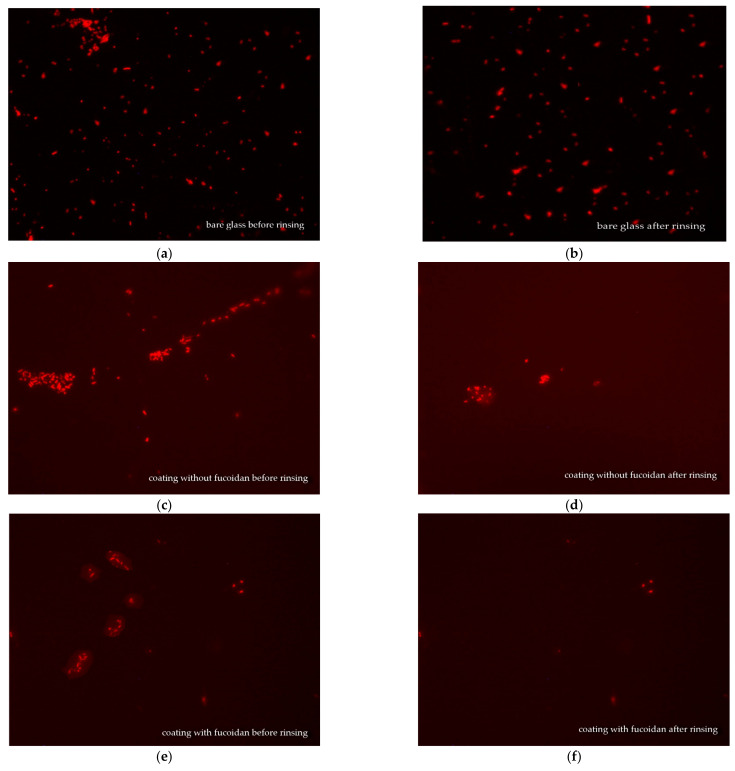
Fluorescence microscopy pictures (×400) of *E. coli* on bare glass (**a**,**b**) and *E. coli* colonies on siloxane coating without fucoidan (**c**,**d**), or on siloxane coating containing 3 wt.% fucoidan (**e**,**f**) before rinsing (**a**,**c**,**e**) and after rinsing in 3 mL of saline (**b**,**d**,**f**).

Figure 5 presents fluorescence microscopy pictures of *E. coli* on bare glass and on siloxane coatings, both with and without fucoidan, before and after rinsing.

The effect of fucoidan on *E. coli* biofilm formation on the same coatings is similar to that on *S. aureus*. The most damaged *E. coli* cell colonies are observed before rinsing on the coating containing fucoidan (Figure 5c,e,f). The *E. coli* biofilm on the bare glass is the same before and after rinsing (Figure 5a,b). The *E. coli* cell colonies are much more scarce after rinsing both the fucoidan-containing (Figure 5e,f) or not siloxane coatings (Figure 5c,d).

This fluorescence microscopy observation depicts the retained antiadhesive properties of the studied medical siloxane coatings in the presence of fucoidan and its boosting effect on *S. aureus* and *E. coli* biofilm formation.

### 3.5. Mode of Antimicrobial Action of the Studied Fucoidan-Containing, Low-Adhesive Siloxane Coatings

**Figure 6:**
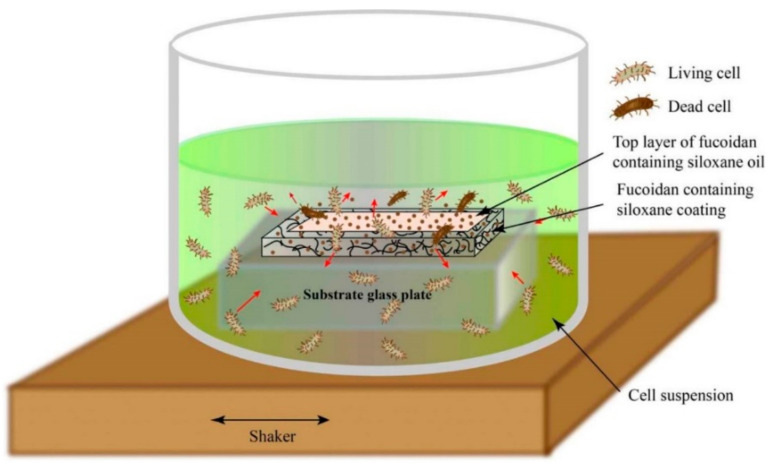
Simplified sketch of the mode of action of fucoidan-containing, low-adhesive siloxane coatings.

All the experimental results give reason to accept the mode of action of fucoidan-containing, low-adhesive siloxane coatings presented in Figure 6.

The growth of both *E. coli* and *S. aureus* cells on the studied fucoidan-containing siloxane coatings is influenced by:

The lack of chemical interaction between the fucoidan and the polysiloxane matrix (proven by XPS analysis, Figure 1 and Figure S3); thus, the fucoidan remains dispersed in the vulcanized coating composition, which contains non-reactive siloxane oil;

The altered vulcanization network in the presence of the dispersed fucoidan, indicated by the altered physical–mechanical surface parameters of the coatings (Table 1, HMV, HIT, and EIT);

The liquid penetrability through this network, evident from the WCAs, measured after 120 s (Table 1, row 2) and the wetting kinetics curves (Figure S2);

The penetrability-conditioned opportunity for fucoidan particles, suspended in the siloxane oil, to leach onto the surface and to form a low-adhesive, biologically active top layer;

The migration ability of the water-soluble fucoidan [14] in the bacterial cell suspension.

Some of the fucoidan particles swell and dissolve in contact with the bacterial suspension, whereas others kill bacterial cells by direct contact on the surface. In this way, the studied low-adhesive siloxane coatings not only make difficult the attachment of bacterial cells but also kill them on contact and release an antimicrobial agent (fucoidan) (Figure 6).

In summary, the observed alteration of bacterial growth and biofilm development in the presence of fucoidan in siloxane coatings could be attributed to the formation of a thin low-adhesive, biologically active surface top layer, similar to that found on antioxidant-containing marine siloxane coatings [10].

## 4. Conclusions

This is the first report on the effect of fucoidan in medical low-adhesive siloxane antimicrobial coatings.

The presence of up to 3–4 wt.% brown algae-derived fucoidan increases their inhibitory effect on bacterial growth, more significantly toward the Gram-positive *S. aureus* than the Gram-negative *Escherichia coli*.

The biological activity of the studied fucoidan-containing, low-adhesive siloxane coatings correlate with the bioadhesion-influencing surface characteristics.

The observed alteration of the bacterial growth and biofilm development in the presence of fucoidan are attributed to the formation of a thin low-adhesive, biologically active top layer consisting of siloxane oil and dispersed water-soluble fucoidan particles.

The experimental results give reason to expect that other non-toxic biologically active natural substances could be utilized in siloxane coatings for improved antimicrobial protection of medical devices.

## Figures and Tables

**Table 1:** Surface physical–chemical characteristics: water contact angle (WCA), surface energy (E), its dispersive (E_d_) and polar (E_p_) components, surface roughness, Ra and Rq, as well as physical–mechanical parameters: dynamic Vicker’s hardness (HMV), indentation hardness (HIT), and indentation elastic modulus (EIT) of the studied coatings: (1)—control, without fucoidan; or containing different amounts fucoidan, wt.%: (2)—1; (3)—2; (4)—3; (5)—5; (6)—8.

Parameter	Coated Glass Sample, No					
	1 Control	2 1 wt.% Fucoidan	3 2 wt.% Fucoidan	4 3 wt.% Fucoidan	5 5 wt.% Fucoidan	6 8 wt.% Fucoidan
WCA, °						
at initial time	106.5 ± 0.21	116.9 ± 0.14	120.2 ± 0.22	117.9 ± 0.70	104.7 ± 4.17	99.7 ± 2.28
after 120 s	100.8 ± 0.35	100.9 ± 0.14	101.4 ± 1.83	103.5 ± 0.21	98.3 ± 4.45	98.3 ± 4.45
E, mN/m						
at initial time	20.0 ± 0.72	19.34 ± 0.15	18.59 ± 0.30	19.39 ± 0.32	20.45 ± 0.50	22.05 ± 0.54
after 120 s	23.31 ± 0.13	23.30 ± 0.31	23.78 ± 0.86	22.19 ± 0.36	24.36 ± 0.74	22.27 ± 2.02
E_d_, mN/m,						
at initial time	20.45 ± 0.02	19.33 ± 0.14	18.42 ± 0.28	19.04 ± 0.26	20.03 ± 0.14	20.91 ± 0.96
after 120 s	22.79 ± 0.10	23.06 ± 0.29	22.89 ± 0.59	16.83 ± 0.28	23.39 ± 0. 12	21.98 ± 1.77
E_p_, mN/m						
at initial time	0.26 ± 0.02	0.01 ± 0.00	0.17 ± 0.02	0.35 ± 0.06	0.41 ± 0.36	1.14 ± 0.54
after 120 s	0.53 ± 0.03	0.23 ± 0.02	0.89 ± 0.26	1.36 ± 0.09	0.98 ± 0.62	5.61 ± 0.02
Ra, nm	0.139	0.163	0.291	0.918	22.316	35.911
Rq, nm	0.217	0.226	0.312	1.316	18.112	40.213
HMV, N/mm^2^	0.120 ± 0.02	0.100 ± 0.02	0.112 ± 0.02	0.117 ± 0.03	0.61 ± 0.02	0.96 ± 0.02
HIT, N/mm^2^	0.361 ± 0.01	0.318 ± 0.02	0.319 ± 0.01	0.422 ± 0.04	0.939 ± 0.02	1.830 ± 0.04
EIT, N/mm^2^	1.66 ± 0.07	1.69 ± 0.04	1.61 ± 0.06	1.90 ± 0.05	9.66 ± 0.09	12.93 ± 0.06

## Data Availability

Not applicable.

## Supplementary Materials

The following supporting information can be downloaded at: https://www.mdpi.com/article/10.3390/ma16103651/s1, Figure S1: Typical structure of fucoidan from brown seaweed consisting of linear backbone build-up by alternating (1 ⟶ 3) and (1 ⟶ 2) linkages and sulfated groups at C4 or C2 (a); or by alternating (1 ⟶ 3) and (1 ⟶ 4) linkages with sulfated groups at C2 or C3 (b); Figure S2: Wetting kinetics, expressed as a contact angle with water (blue), ethylene glycol (green), and n-hexadecane of silicon coating-covered glass samples: (sample 1)—control without fucoidan, or; containing different amounts of fucoidan, wt.%: (sample 2)—1; (sample 3)—2; sample (4)—3; (sample 5)—5; (sample 6)—8; Figure S3: Deconvoluted C1s (a), O1s (b), Si2p (c), and S2p (d) peaks of cross-linked siloxane elastomer (curves 1), cross-linked siloxane containing 5 wt.% fucoidan (curves 2), and (c)—fucoidan (curves 3).
