# Supplementary material for: Fucoidan-Containing, Low-Adhesive Siloxane Coatings for Medical Applications: Inhibition of Bacterial Growth and Biofilm Development
Source: Materials (Basel). 2023 May 10;16(10):3651. doi: 10.3390/ma16103651 (PMC10222722; doi:10.3390/ma16103651)
Supplement: Supplementary file 1 [file materials-16-03651-s001.zip › Vladkova_Figure S 3_Deconvoluted XPS.pdf]

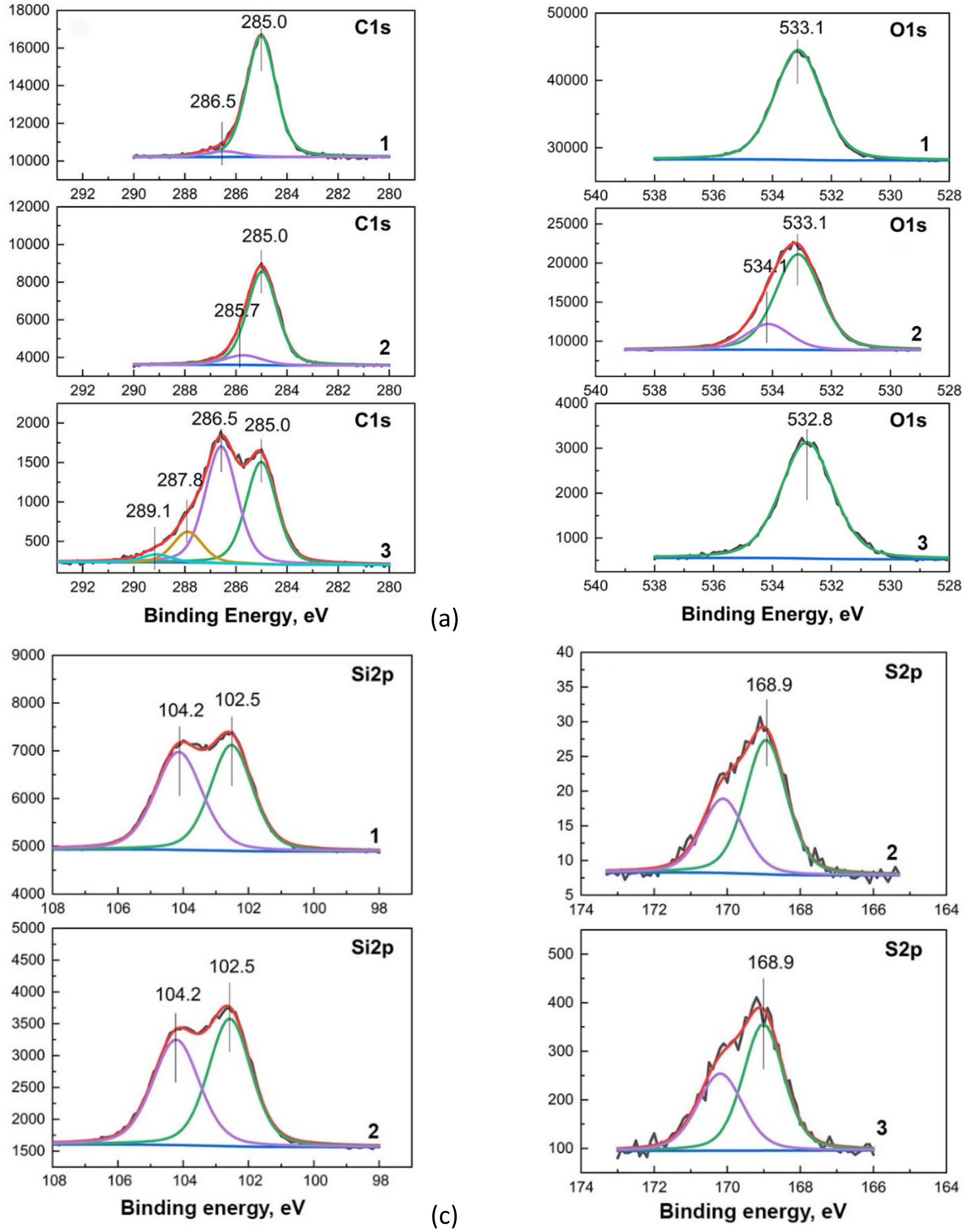

Figure S3. Deconvoluted C1s (a), O1s (b), Si2p (c) and S2p (d) peaks of: cross-linked siloxane elastomer (curves 1); cross-linked siloxane containing 5 wt. % fucoidan (curves 2) and (c) - fucoidan (curves 3)
