# Supplementary material for: Fucoidan-Containing, Low-Adhesive Siloxane Coatings for Medical Applications: Inhibition of Bacterial Growth and Biofilm Development
Source: Materials (Basel). 2023 May 10;16(10):3651. doi: 10.3390/ma16103651 (PMC10222722; doi:10.3390/ma16103651)
Supplement: Supplementary file 1 [file materials-16-03651-s001.zip › Vladkova_Figure S 1_Typical structure of fucoidan from brown seaweeds.pdf]

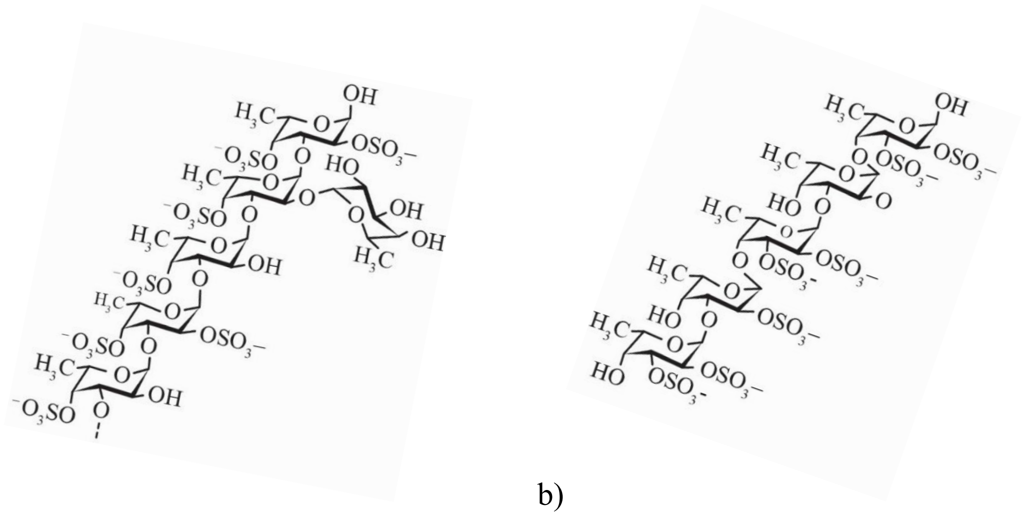

a) b)

Figure S1 Typical structure of fucoidan from brown seaweeds consisting of linear backbone build up by alternating (1 $\rightarrow$ 3) and (1 $\rightarrow$ 2) linkages and sulphated groups at C4 or C2 (a); or by alternating (1 $\rightarrow$ 3) and (1 $\rightarrow$ 4) linkages with sulphated groups at C2 or C3 (b)
