# Supplementary material for: Fucoidan-Containing, Low-Adhesive Siloxane Coatings for Medical Applications: Inhibition of Bacterial Growth and Biofilm Development
Source: Materials (Basel). 2023 May 10;16(10):3651. doi: 10.3390/ma16103651 (PMC10222722; doi:10.3390/ma16103651)
Supplement: Supplementary file 1 [file materials-16-03651-s001.zip › Vladkova_Figure S 2_Wetting kynetics.pdf]

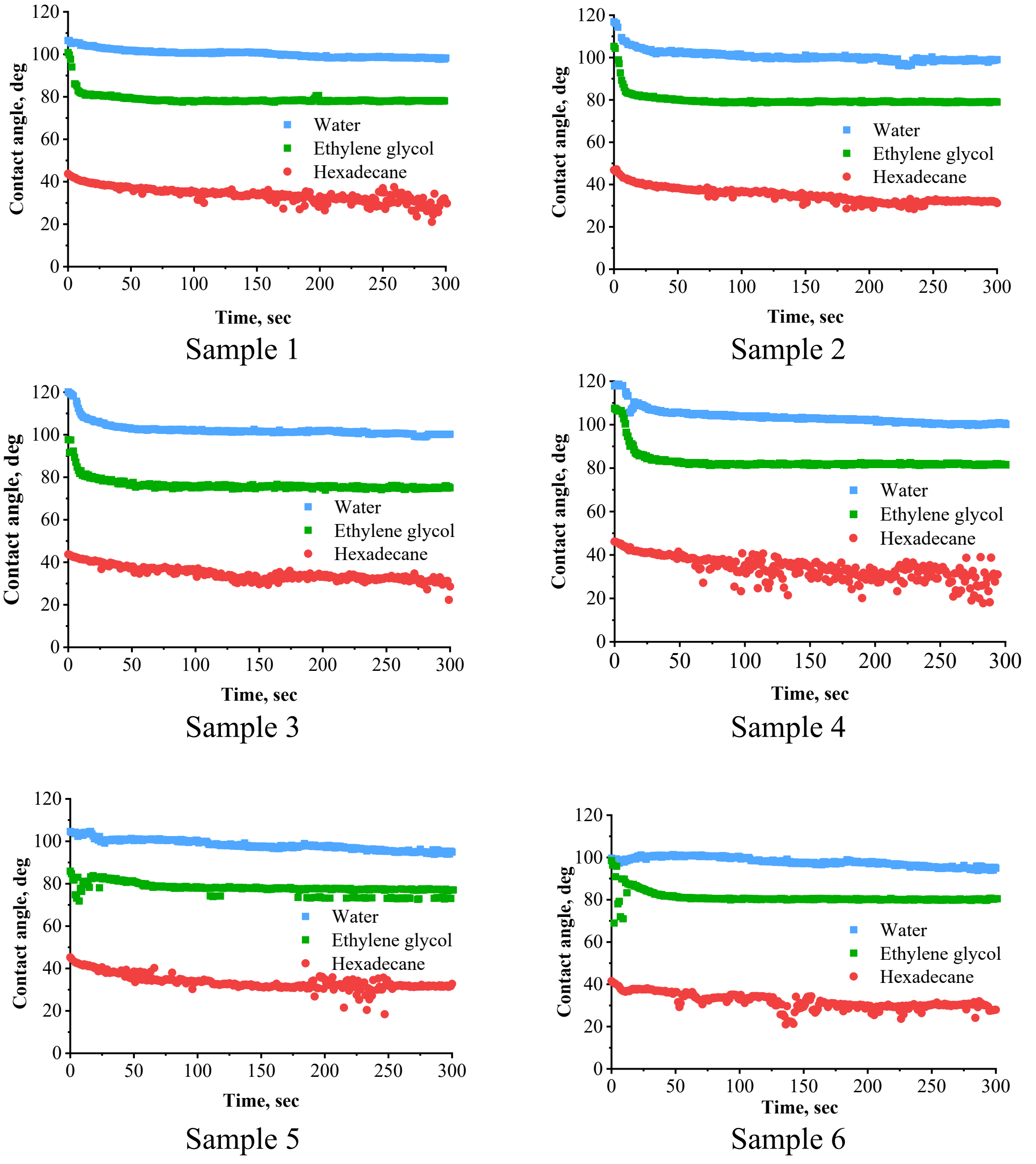

Figure S2. Wetting kinetics, expressed as a contact angle with water (blue), ethylene glycol (green) and n-hexadecan of silicon coatings covered glass samples: (sample 1) - control without fucoidan or; containing different amounts of fucoidan, wt. %: (sample 2) – 1; (sample 3) - 2; sample (4) - 3; (sample 5) - 5; (sample 6) – 8.
